# Investigation of surface roughness caused by different irrigation solutions on Biodentine and NeoPutty MTA used in perforation repair

**DOI:** 10.34172/joddd.025.43857

**Published:** 2025-09-30

**Authors:** Zeynep Toprak, Faruk Öztekin

**Affiliations:** ^1^Department of Endodontics, Mardin Oral and Dental Health Center, Mardin, Turkey; ^2^Department of Endodontics, Faculty of Dentistry, Fırat University, Elazığ, Turkey

**Keywords:** Biodentine, Ethylenediaminetetraacetic acid, Maleic acid, NeoPutty MTA, Surface roughness

## Abstract

**Background.:**

This study aimed to investigate the effect of different irrigation solutions on the surface roughness of NeoPutty MTA and Biodentine materials used as perforation repair materials.

**Methods.:**

Thirty-two Teflon blocks were divided into M (NeoPutty MTA) and B (Biodentine) groups, which were further divided into maleic acid (n=8) and ethylenediaminetetraacetic acid (EDTA) (n=8) groups. The surface roughness of all the samples was determined by scanning with an atomic force microscopy (AFM) device before and after soaking in 7% maleic acid and 17% EDTA solutions. For data analysis, the Mann-Whitney U test was used to compare two independent groups, and the Wilcoxon signed rank test was used for dependent groups.

**Results.:**

According to the findings of the study, no significant difference was found between the initial roughness of NeoPutty MTA and Biodentine materials. However, EDTA solution produced statistically significant surface roughness in Biodentine material, while maleic acid solution produced statistically significant surface roughness in NeoPutty MTA material.

**Conclusion.:**

Further studies are necessary to investigate the effects of physicochemical changes induced by irrigation solutions in repair materials on bacterial adhesion and restorative adhesive procedures.

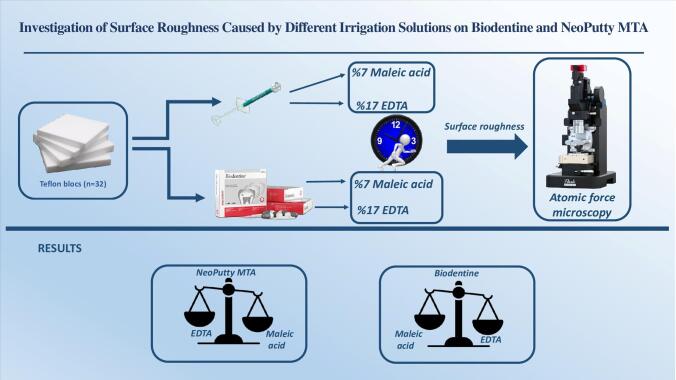

## Introduction

 When the root canal is prepared using either hand or rotary instruments, the dentin surface is significantly disrupted, resulting in the formation of a layer,^1^ composed of both organic and inorganic debris produced during root canal preparation, collectively referred to as the smear layer.^2^ Complete removal of the smear layer is considered desirable.^3^

 Studies have shown that the most effective method for removing the smear layer is the combined use of sodium hypochlorite (NaOCl), an organic tissue solvent, and ethylenediaminetetraacetic acid (EDTA), an inorganic tissue solvent.^4^ EDTA, commonly used in endodontic irrigation, chemically softens root canal dentin, dissolves the smear layer, and increases dentin permeability.^5^ However, its smear layer removal capacity should be evaluated alongside its potential toxic and erosive effects.^6^

 Maleic acid is a mild organic acid used in adhesive dentistry for surface conditioning without rinsing.^7^ It also exhibits antibacterial properties, which are attributed to a reduction in intracellular pH. This occurs when protons are released from undissociated molecules within the cytoplasm, leading to decreased activity of essential enzymes.^8^

 Root canal perforations are undesirable complications that can occur at any stage of root canal treatment, potentially leading to irritation and loss of periodontal tissues.^9,10^ Although perforations may result from resorption or caries, the majority are of iatrogenic origin, arising during various stages of treatment.^10,11^ Iatrogenic perforations often result from inadequate knowledge of root canal anatomy or failure to consider anatomical variations.^11^

 The worst prognosis in endodontic perforations has been observed in furcation perforations that occur when trying to open an access cavity with an incorrectly angled bur, during post space preparation, or when trying to find calcified root canal orifices.^12^

 Mineral trioxide aggregate (MTA), known for its excellent sealing ability and high biocompatibility, is widely used in various endodontic procedures, including perforation repair.^13^ However, despite its clinical advantages, MTA also presents drawbacks such as prolonged setting time and challenging handling properties.^14^

 To address the limitations of MTA, such as difficult handling, prolonged setting time, and high cost, Biodentine was introduced in 2010 as a new bioceramic material. Compared to MTA, Biodentine offers the advantages of shorter setting time and lower cost.^14^

 The handling difficulties associated with calcium silicate cements have led to the development of the concept that a premixed formulation could simplify clinical use while ensuring reproducibility of the precise liquid-to-powder ratio.^15^ NeoPutty (NuSmile, Houston, TX, USA) is a premixed, bioactive, tricalcium silicate-based cement that, according to the manufacturer, addresses these challenges.^16,17^

 Irrigating agents used in root canal treatment remove the smear layer, expose dentinal tubules, and increase surface roughness.^18^ A rougher substrate surface may promote bacterial adhesion and biofilm formation.^19^

 In recent years, atomic force microscopy (AFM), which has been widely applied in dental materials research, has enabled the acquisition of nanometric topographic images of surface roughness.^20^ AFM combines principles from the mechanical profilometer, which detects forces via mechanical springs, and the scanning tunnelling microscope (STM), which uses piezoelectric transducers for scanning. However, AFM allows more precise measurements than a conventional profilometer. The instrument characterises specimen morphology and provides quantitative data on parameters such as surface roughness and height distribution.^21,22^

 Therefore, this study used AFM to investigate the changes in surface roughness induced by EDTA and maleic acid irrigation solutions on NeoPutty MTA and Biodentine, two materials commonly used in perforation repair.

## Methods

 The required sample size (n = 32) was calculated using G*Power software based on an effect size of 0.80, a power of 95%, and a significance level of 0.05 for one-way ANOVA. Each of the four groups consisted of 8 samples.

 Standardized grooves measuring 2 mm in diameter and 2 mm in depth were prepared at the center of 32 Teflon blocks used in this study. The blocks were then divided into two main groups according to the repair material: M (NeoPutty MTA) and B (Biodentine). Each main group was further subdivided based on the irrigation solution into maleic acid and EDTA groups.

**Figure 1:**
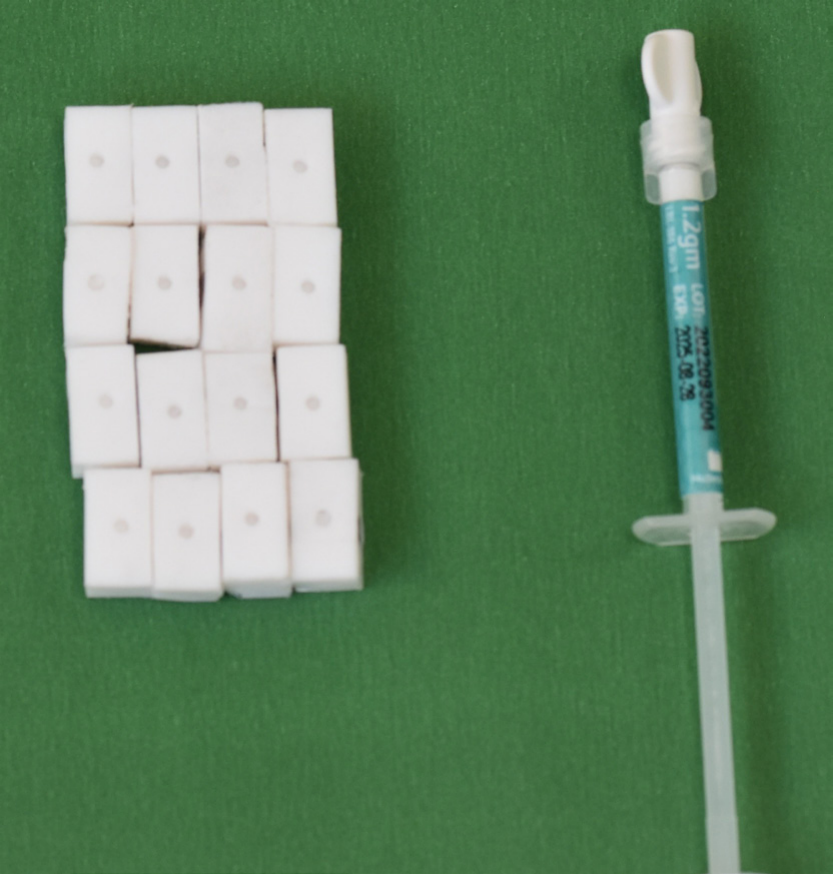
NeoPutty MTA (Avalon Biomed, USA). The premixed, calcium silicate-based endodontic material was used in this study

**Figure 2:**
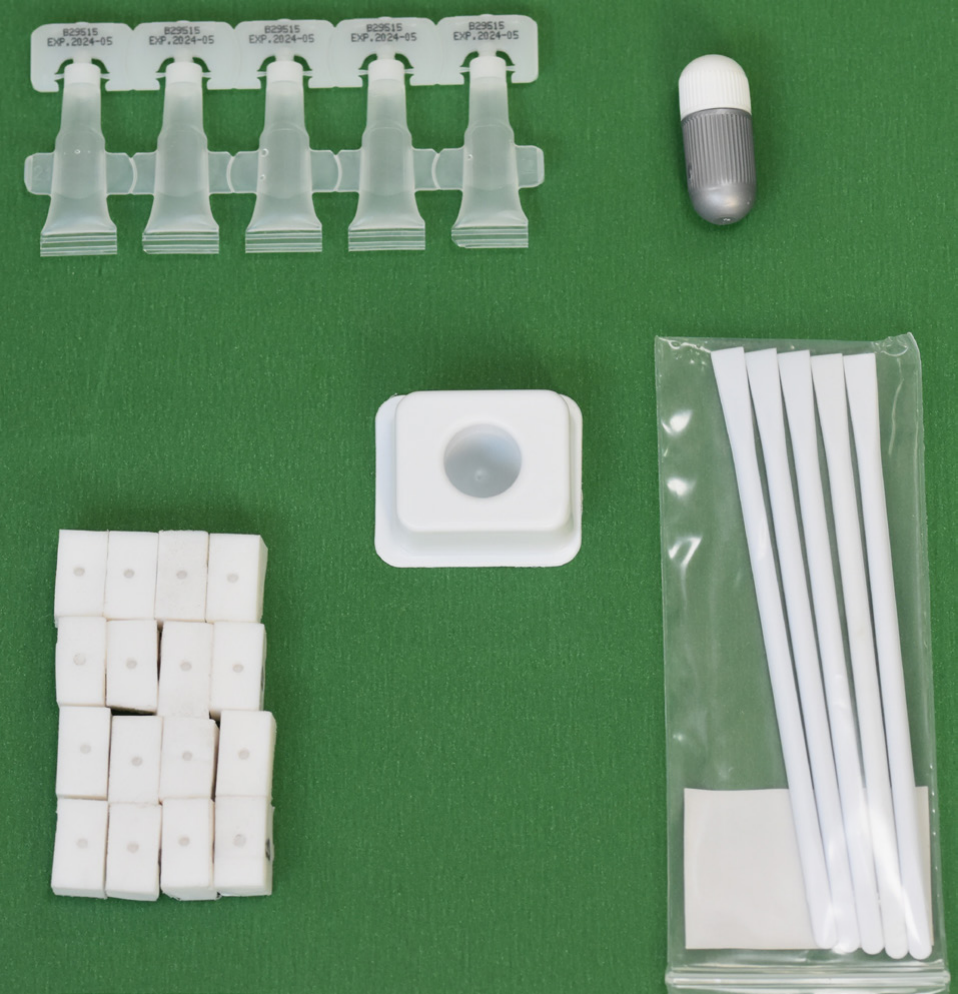
Biodentine. The calcium silicate-based endodontic material was used in this study

 NeoPutty MTA (NuSmile Inc., Houston, TX, USA) (Figure 1) and Biodentine (Septodont, Niederkassel, Germany) (Figure 2) were applied onto the Teflon blocks and condensed following the manufacturers’ instructions. The samples were then covered with moistened gauze and incubated in a humidified oven at 37°C for 7 days to allow setting.

 After the storage period, surface irregularities of the NeoPutty MTA and Biodentine samples were smoothened using sequentially finer grades of water-resistant silicon carbide sandpaper (Shor International Corporation, Mt. Vernon, NY, USA) under continuous distilled water irrigation (grits: 500, 800, 1000, and 1200). Final polishing was performed with 0.1-µm alumina suspension polishing paste (Ultra-Sol R, Eminess Technologies Inc., Monroe, NC, USA) using felt discs.

**Figure 3:**
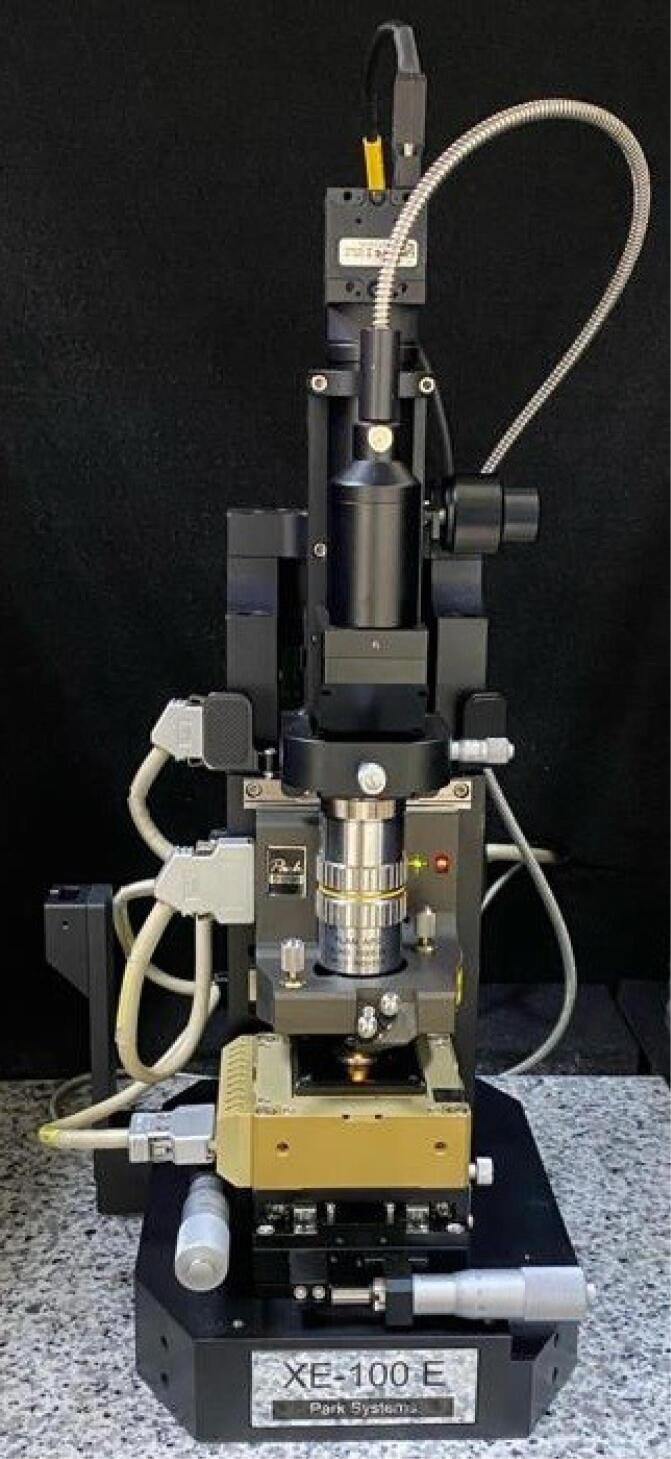
XE-100E atomic force microscope (Park Systems, Suwon, South Korea). The device was used to measure the surface roughness of the tested materials

 Baseline surface roughness measurements were taken for all samples before exposure to any chelating agents, using a PARK SYSTEM 100 XE AFM (Figure 3) in contact mode.

 After the measurement, NeoPutty MTA and Biodentine samples were divided into two groups and kept in 7% maleic acid (Merck Millipore, Germany) and 17% EDTA (Imicryl, Konya, Turkey) solutions for 1 minute each. After completing the 1-minute waiting time, all the samples were removed from the irrigation solutions, washed under running distilled water, and kept in a humid environment at 37 °C for 48 hours. The final surface roughness was determined by scanning again with the AFM device.

 For data analysis, the Mann-Whitney U test was used to compare two independent groups, and the Wilcoxon signed-rank test was used for dependent groups.

## Results

**Figure 4:**
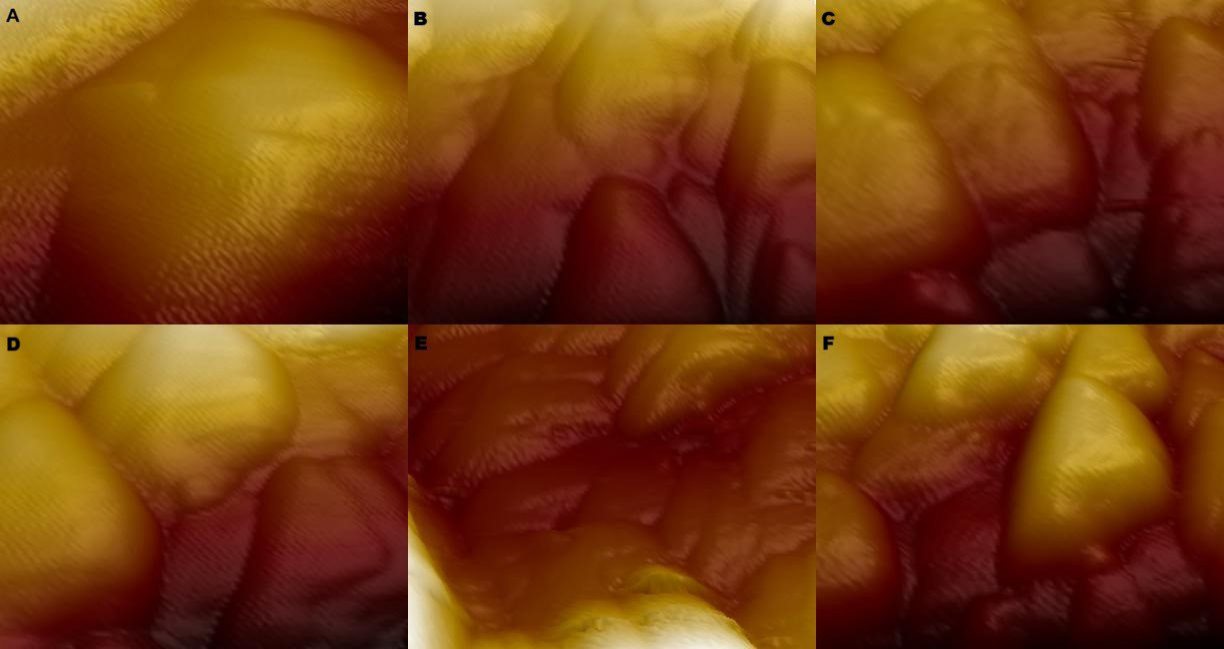
(A) AFM image of the initial surface roughness of Biodentine material. (B) AFM image of the initial surface roughness of NeoPutty MTA material. (C) AFM image of surface roughness of Biodentine material after maleic acid solution exposure. (D) AFM image of the surface roughness of Biodentine material after EDTA solution exposure. (E) AFM image of the surface roughness of NeoPutty MTA material after maleic acid solution exposure. (F) AFM image of the surface roughness of NeoPutty MTA material after EDTA solution exposure

 To determine the baseline surface roughness of NeoPutty MTA and Biodentine samples before treatment with 17% EDTA and 7% maleic acid, 16 samples of each material were prepared on 32 Teflon blocks. Surface roughness measurements were performed using an AFM in contact mode before irrigation (Figure 4).

 There was no significant difference in the initial surface roughness between the Biodentine (n = 16) and NeoPutty MTA (n = 16) groups (*P* = 0.559).

Table 1 presents the median surface roughness values of Biodentine and NeoPutty MTA samples, subdivided into EDTA and maleic acid groups, both before and after immersion in the solutions.

**Table 1:** Comparison of surface roughness values before and after treatment. Statistical analysis was performed using the Wilcoxon signed-rank test

		**Pre-processing **	**Postprocessing**	***P*** *
Biodentine	Maleic acid (n = 8)	162.5 (74.5–356.33)	294.27 (127.9–520.94)	0.128
	EDTA (n = 8)	125.72 (57.67–268.5)	393.53 (252.25–589.79)	0.028
NeoPUTTY MTA	Maleic acid (n = 8)	118.35 (51.83–249)	220.88 (156.88–475)	0.017
	EDTA (n = 8)	192.64 (40.9–483.07)	154.48 (119.16–549.8)	0.575

*P* < 0.05 was considered statistically significant. * Wilcoxon sign-rank test.

 According to these findings, maleic acid solution did not cause a significant change in the surface roughness of Biodentine (*P* = 0.128). However, the surface roughness of Biodentine after exposure to EDTA solution was significantly higher than the initial value (*P* = 0.028).

 In contrast, the initial surface roughness of NeoPutty MTA was significantly lower than that measured after exposure to maleic acid solution (*P* = 0.017). Additionally, exposure to EDTA solution did not result in a significant change in the surface roughness of NeoPutty MTA (*P* = 0.575).

Table 2 presents the median roughness values induced by maleic acid and EDTA on NeoPutty MTA and Biodentine.

 According to these data, maleic acid solution induced comparable surface roughness in both NeoPutty MTA and Biodentine materials. However, EDTA solution caused greater roughening in Biodentine compared to NeoPutty MTA.

**Table 2:** Comparison of surface roughness values after treatment between groups. Statistical analysis was performed using the Mann-Whitney U test

	**NeoPUTTY MTA**	**Biodentine**	***P*** *
Maleic acid	220.88 (156.88–475)	294.27 (127.9–520.945)	0.463
EDTA	154.48 (119.16–549.8)	393.53 (252.25–589.79)	0.043

*P* < 0.05 was considered statistically significant. *Mann-Whitney U test.

## Discussion

 Endodontic perforations are serious complications that hinder treatment procedures and negatively affect the prognosis of the tooth.^23,24^ MTA is commonly preferred for perforation repair due to its biocompatibility, sealing ability, and high clinical success rate.^10^ However, recent studies have indicated that the calcium silicate-based material Biodentine may be more effective than MTA in perforation closure.^25,26^

 In endodontic treatment, irrigation is the only method to reach areas of the root canal walls inaccessible to mechanical instruments. Irrigation also plays a crucial role in removing microorganisms, tissue debris, and dentin chips through a flushing mechanism.^27^ However, irrigating agents can induce structural changes in the tissues they contact.^28^

 Based on this background, the present study evaluated the surface roughness caused by chelating agents and acids used in root canal treatment on materials employed for perforation repair.

 In this study, a comparison of the effects of EDTA and maleic acid solutions on the surface roughness of NeoPutty MTA and Biodentine revealed that EDTA caused the greatest roughening of Biodentine. In contrast, maleic acid resulted in the highest roughness in NeoPutty MTA.

 Previous studies have demonstrated that AFM provides more detailed surface roughness measurements compared to profilometers.^20,29^ Therefore, AFM was employed in the present study to achieve a more precise analysis of surface topography.

 In addition to sodium hypochlorite (NaOCl), decalcifying agents are necessary to remove both the organic and inorganic components of the smear layer formed during root canal preparation. Among these, EDTA is commonly used to effectively remove the inorganic component from canal walls.^30^

 In a study by Kaushal et al,^31^ 17% EDTA, 10% citric acid, and 7% maleic acid were each applied for 1 minute. The results indicated that both 7% maleic acid and 10% citric acid were equally effective in removing the smear layer from the coronal, middle, and apical thirds of the root canal; however, 7% maleic acid demonstrated superior efficacy in the apical third compared to 10% citric acid.

 Ballal et al^32^ investigated the in vitro antimicrobial activity of 7% maleic acid and 17% EDTA solutions against endodontic pathogens, finding that both solutions exhibited comparable antimicrobial effects.

 Based on the literature, maleic acid, which effectively dissolves the smear layer, is a promising irrigation agent in root canal treatment. Therefore, 7% maleic acid was considered a potential alternative to EDTA and was applied for 1 minute in this study.

 A previous study^33^ examining the effect of EDTA on the surface roughness of Biodentine reported a significant increase in roughness following exposure, consistent with the findings of the present study.

 Furthermore, an AFM-based investigation of dentin surface roughness caused by irrigation solutions^34^ found that the roughness increase induced by EDTA was similar to that observed on NeoPutty MTA and Biodentine in the current study. This suggests that EDTA may exert comparable effects on dental materials rich in calcium and phosphorus, possibly through its chelating action by dissociating calcium ions, similar to its effect on root canal dentin. In a study investigating the effects of EDTA on the hydration mechanism of MTA using scanning electron microscopy and energy-dispersive X-ray spectroscopy,^35^ MTA samples treated with EDTA lacked a crystalline structure, and their Ca/Si ratio was markedly lower than that of samples treated with distilled water or normal saline solution, indicating structural alterations. When these findings are considered alongside other studies in the literature and the results of the present study, it can be inferred that perforation repair materials exposed to chelating agents and acids should be rinsed with distilled water after use, or their contact time should be limited to an optimal duration.

 A study evaluating the effects of various finishing and polishing techniques on the surface roughness and microhardness of dental materials^36^ reported that the correlation between these two properties depends on both the method and the material used. For Dyract XP and Beautifil II, a negative correlation was observed—meaning that increased surface roughness was associated with decreased microhardness values.

 Another study evaluating the effects of bleaching agents on enamel showed that increased surface roughness was associated with decreased surface microhardness.^37^

 In the present study, both EDTA and maleic acid were found to increase the surface roughness of NeoPutty MTA and Biodentine. Considering the findings of the two aforementioned studies, the observed negative correlation between increased surface roughness and decreased microhardness highlights the need for appropriate measures to minimize surface roughness in dental materials. Nevertheless, further research is required to determine whether this correlation also applies to the materials tested in the present study.

 The changes caused by chelating agents and acids used in root canal treatment on the surface of perforation repair materials are important. Rinsing with distilled water after irrigation does not prevent the changes that occur on the surface of the solution during contact with the material. Silicate cements used in perforation repair may be affected by chelating solutions and acids used in irrigation, and severe deterioration may occur in these materials. In addition, increased surface roughness may create a retentive surface for microorganisms.^38,39^ Several studies investigating the relationship between surface roughness and bacterial adhesion have reported a positive correlation between these two parameters.^40,41^ However, these findings are not always consistent; some studies have suggested no significant relationship between surface roughness and bacterial adhesion.^42^ Indeed, a study by Azam et al demonstrated that bacterial adhesion is influenced not only by surface topography but also by several factors, including particle size, chemical composition, and surface wettability of the material.^43^

 On the other hand, surface roughness is directly related to the bonding interface of restorative materials.^44^ However, excessive roughness may hinder the penetration of adhesives into the material surface, negatively affecting bond strength.^45^ In a study investigating the bond strength of Biodentine after surface treatment with different adhesives,^46^ specimens treated with more aggressive acids exhibited reduced bond strength. Similar findings have also been reported in studies on MTA.^47,48^ In this context, the effects of irrigation solutions on surface morphology and their implications for bonding performance should be carefully evaluated.

 The irrigation solutions used in this study affected the surface roughness of the repair materials at different rates. For all these reasons, in cases where calcium silicate cements are to be used in perforation repairs, irrigation solutions that will not affect or minimally affect the surface structure of these cements should be preferred.

 Further studies are required to determine the effects of physicochemical changes of irrigation solutions on bacterial adhesion and restorative adhesive procedures. In addition, within the limitations of the present study, many other variables in the clinical environment, such as blood, tissue, and body temperature, may alter the effects of the investigated agents in the root canal system. However, these clinical conditions could not be simulated in the present study. Further research is needed to understand the effects of maleic acid and EDTA solutions on NeoPutty MTA and Biodentine materials.

## Conclusion

 Acids and chelating agents generally increase the surface roughness of dental materials and dental tissues. In clinical use, it should be taken into consideration that the materials to be used in perforation repair may be affected by irrigation solutions, and deterioration may occur on their surfaces. In cases where calcium silicate cements will be used in perforation repair, irrigation solutions that will not affect or minimally affect the surface structure of these cements should be preferred. Therefore, further studies are required to determine the effects of physicochemical changes of irrigation solutions on bacterial adhesion and restorative adhesive procedures.

## Competing Interests

 The authors declare no conflicts of interest.

## Ethical Approval

 This study did not involve human participants or animals, and therefore, ethical approval was not required.

## References

[1] Violich DR, Chandler NP (2010). The smear layer in endodontics - a review. Int Endod J.

[2] McComb D, Smith DC (1975). A preliminary scanning electron microscopic study of root canals after endodontic procedures. J Endod.

[3] Gulabivala K, Patel B, Evans G, Ng YL (2005). Effects of mechanical and chemical procedures on root canal surfaces. Endod Topics.

[4] Yamada RS, Armas A, Goldman M, Lin PS (1983). A scanning electron microscopic comparison of a high-volume final flush with several irrigating solutions: part 3. J Endod.

[5] Cruz-Filho AM, Sousa-Neto MD, Savioli RN, Silva RG, Vansan LP, Pécora JD (2011). Effect of chelating solutions on the microhardness of root canal lumen dentin. J Endod.

[6] Serper A, Calt S, Dogan AL, Guc D, Ozçelik B, Kuraner T (2001). Comparison of the cytotoxic effects and smear layer removing capacity of oxidative potential water, NaOCl and EDTA. J Oral Sci.

[7] Fu B, Yuan J, Qian W, Shen Q, Sun X, Hannig M (2004). Evidence of chemisorption of maleic acid to enamel and hydroxyapatite. Eur J Oral Sci.

[8] Olasupo NA, Fitzgerald DJ, Narbad A, Gasson MJ (2004). Inhibition of Bacillus subtilis and Listeria innocua by nisin in combination with some naturally occurring organic compounds. J Food Prot.

[9] Meister F Jr, Lommel TJ, Gerstein H, Davies EE (1979). Endodontic perforations which resulted in alveolar bone loss Report of five cases. Oral Surg Oral Med Oral Pathol.

[10] Main C, Mirzayan N, Shabahang S, Torabinejad M (2004). Repair of root perforations using mineral trioxide aggregate: a long-term study. J Endod.

[11] Alhadainy HA (1994). Root perforations A review of literature. Oral Surg Oral Med Oral Pathol.

[12] Aguirre R, ElDeeb ME, ElDeeb ME (1986). Evaluation of the repair of mechanical furcation perforations using amalgam, gutta-percha, or indium foil. J Endod.

[13] Dong X, Xu X (2023). Bioceramics in endodontics: updates and future perspectives. Bioengineering (Basel).

[14] Kaur M, Singh H, Dhillon JS, Batra M, Saini M (2017). MTA versus Biodentine: review of literature with a comparative analysis. J Clin Diagn Res.

[15] Persson C, Engqvist H (2011). Premixed calcium silicate cement for endodontic applications: injectability, setting time and radiopacity. Biomatter.

[16] Sun Q, Meng M, Steed JN, Sidow SJ, Bergeron BE, Niu LN (2021). Manoeuvrability and biocompatibility of endodontic tricalcium silicate-based putties. J Dent.

[17] Lozano-Guillén A, López-García S, Rodríguez-Lozano FJ, Sanz JL, Lozano A, Llena C (2022). Comparative cytocompatibility of the new calcium silicate-based cement NeoPutty versus NeoMTA Plus and MTA on human dental pulp cells: an in vitro study. Clin Oral Investig.

[18] Eldeniz AU, Erdemir A, Belli S (2005). Effect of EDTA and citric acid solutions on the microhardness and the roughness of human root canal dentin. J Endod.

[19] Bollen CM, Lambrechts P, Quirynen M (1997). Comparison of surface roughness of oral hard materials to the threshold surface roughness for bacterial plaque retention: a review of the literature. Dent Mater.

[20] Kakaboura A, Fragouli M, Rahiotis C, Silikas N (2007). Evaluation of surface characteristics of dental composites using profilometry, scanning electron, atomic force microscopy and gloss-meter. J Mater Sci Mater Med.

[21] Meyer E (1992). Atomic force microscopy. Prog Surf Sci.

[22] Binnig G, Gerber C, Stoll E, Albrecht TR, Quate CF (1987). Atomic resolution with atomic force microscope. Europhys Lett.

[23] Sinai IH (1977). Endodontic perforations: their prognosis and treatment. J Am Dent Assoc.

[24] Nagpal R, Manuja N, Pandit IK, Rallan M. Surgical management of iatrogenic perforation in maxillary central incisor using mineral trioxide aggregate. BMJ Case Rep 2013;2013. doi: 10.1136/bcr-2013-200124. PMC3736630 23845686

[25] Ajas A, Anulekh B, Nasil S, Thaha KA, Mary VJ (2018). Comparative evaluation of sealing ability of Biodentine and white MTA-Angelus as furcation repair materials: a dye extraction study. Int J Oral Care Res.

[26] Aggarwal V, Singla M, Miglani S, Kohli S (2013). Comparative evaluation of push-out bond strength of ProRoot MTA, Biodentine, and MTA Plus in furcation perforation repair. J Conserv Dent.

[27] Haapasalo M, Shen Y, Qian W, Gao Y (2010). Irrigation in endodontics. Dent Clin North Am.

[28] Chubb DW (2019). A review of the prognostic value of irrigation on root canal treatment success. Aust Endod J.

[29] Karatas O, Gul P, Gündoğdu M, Iskenderoglu DT (2020). An evaluation of surface roughness after staining of different composite resins using atomic force microscopy and a profilometer. Microsc Res Tech.

[30] Basrani B, Haapasalo M (2012). Update on endodontic irrigating solutions. Endod Topics.

[31] Kaushal R, Bansal R, Malhan S (2020). A comparative evaluation of smear layer removal by using ethylenediamine tetraacetic acid, citric acid, and maleic acid as root canal irrigants: an in vitro scanning electron microscopic study. J Conserv Dent.

[32] Ballal NV, Yegneswaran PP, Mala K, Bhat KS (2011). In vitro antimicrobial activity of maleic acid and ethylenediaminetetraacetic acid on endodontic pathogens. Oral Surg Oral Med Oral Pathol Oral Radiol Endod.

[33] Ulusoy Öİ, Paltun YN, Ulusoy N (2017). Etilendiamin tetraasetik asit ve etidronik asitin Biodentine yüzey pürüzlülüğü üzerine etkisi: in vitro. Acta Odontol Turc.

[34] Hu X, Ling J, Gao Y (2010). Effects of irrigation solutions on dentin wettability and roughness. J Endod.

[35] Lee YL, Lin FH, Wang WH, Ritchie HH, Lan WH, Lin CP (2007). Effects of EDTA on the hydration mechanism of mineral trioxide aggregate. J Dent Res.

[36] Aydın EG, Özalp N (2021). Which finishing and polishing technique is more effective for surface roughness and microhardness?. Cumhur Dent J.

[37] Pinto CF, Oliveira R, Cavalli V, Giannini M (2004). Peroxide bleaching agent effects on enamel surface microhardness, roughness and morphology. Braz Oral Res.

[38] Quirynen M, Bollen CM (1995). The influence of surface roughness and surface-free energy on supra- and subgingival plaque formation in man A review of the literature. J Clin Periodontol.

[39] Tang L, Pillai S, Revsbech NP, Schramm A, Bischoff C, Meyer RL (2011). Biofilm retention on surfaces with variable roughness and hydrophobicity. Biofouling.

[40] Aykent F, Yondem I, Ozyesil AG, Gunal SK, Avunduk MC, Ozkan S (2010). Effect of different finishing techniques for restorative materials on surface roughness and bacterial adhesion. J Prosthet Dent.

[41] Kozmos M, Virant P, Rojko F, Abram A, Rudolf R, Raspor P (2021). Bacterial adhesion of Streptococcus mutans to dental material surfaces. Molecules.

[42] Bunz O, Diekamp M, Bizhang M, Testrich H, Piwowarczyk A (2024). Surface roughness associated with bacterial adhesion on dental resin-based materials. Dent Mater J.

[43] Azam MT, Khan AS, Muzzafar D, Faryal R, Siddiqi SA, Ahmad R (2015). Structural, surface, in vitro bacterial adhesion and biofilm formation analysis of three dental restorative composites. Materials.

[44] Rosentritt M, Preis V, Behr M, Sereno N, Kolbeck C (2015). Shear bond strength between veneering composite and PEEK after different surface modifications. Clin Oral Investig.

[45] Jennings CW (1972). Surface roughness and bond strength of adhesives. J Adhes.

[46] Çolak H, Tokay U, Uzgur R, Uzgur Z, Ercan E, Hamidi MM (2016). The effect of different adhesives and setting times on bond strength between Biodentine and composite. J Appl Biomater Funct Mater.

[47] Tyagi N, Chaman C, Tyagi SP, Singh UP, Sharma A (2016). The shear bond strength of MTA with three different types of adhesive systems: an in vitro study. J Conserv Dent.

[48] Atabek D, Sillelioğlu H, Olmez A (2012). Bond strength of adhesive systems to mineral trioxide aggregate with different time intervals. J Endod.

